# Supplementary material for: Improved Bladder Tumor RNA Isolation from Archived Tissues Using Methylene Blue for Normalization, Multiplex RNA Hybridization, Sequencing and Subtyping
Source: Int J Mol Sci. 2022 Sep 6;23(18):10267. doi: 10.3390/ijms231810267 (PMC9499321; doi:10.3390/ijms231810267)
Supplement: Supplementary file 1 [file ijms-23-10267-s001.zip › ijms-1839240-supplementary.pdf]

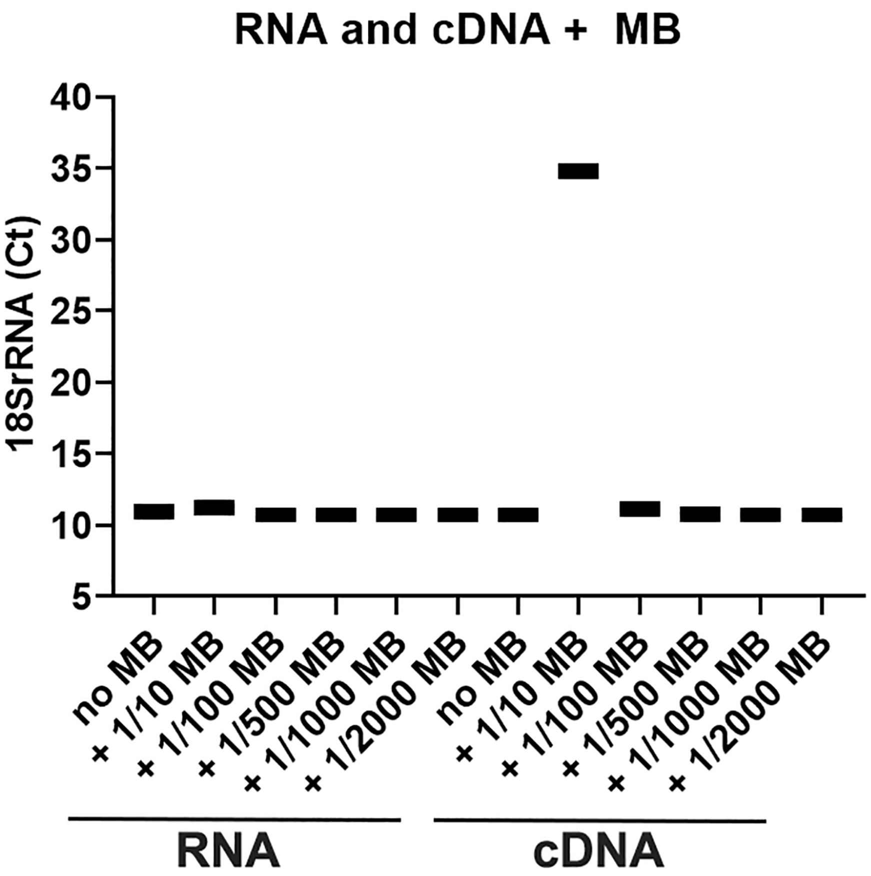

Figure S1. MB non-interference with RNA and cDNA using qPCR. Bar graph shows *18SrRNA* qPCR Ct values (Y-axis) from purified RNA incubated with (+) MB testing a series of dilutions (X-axis) and incubated with cDNA prior to qPCR testing a series of dilutions. RNA and cDNA (-) MB was used as a control.

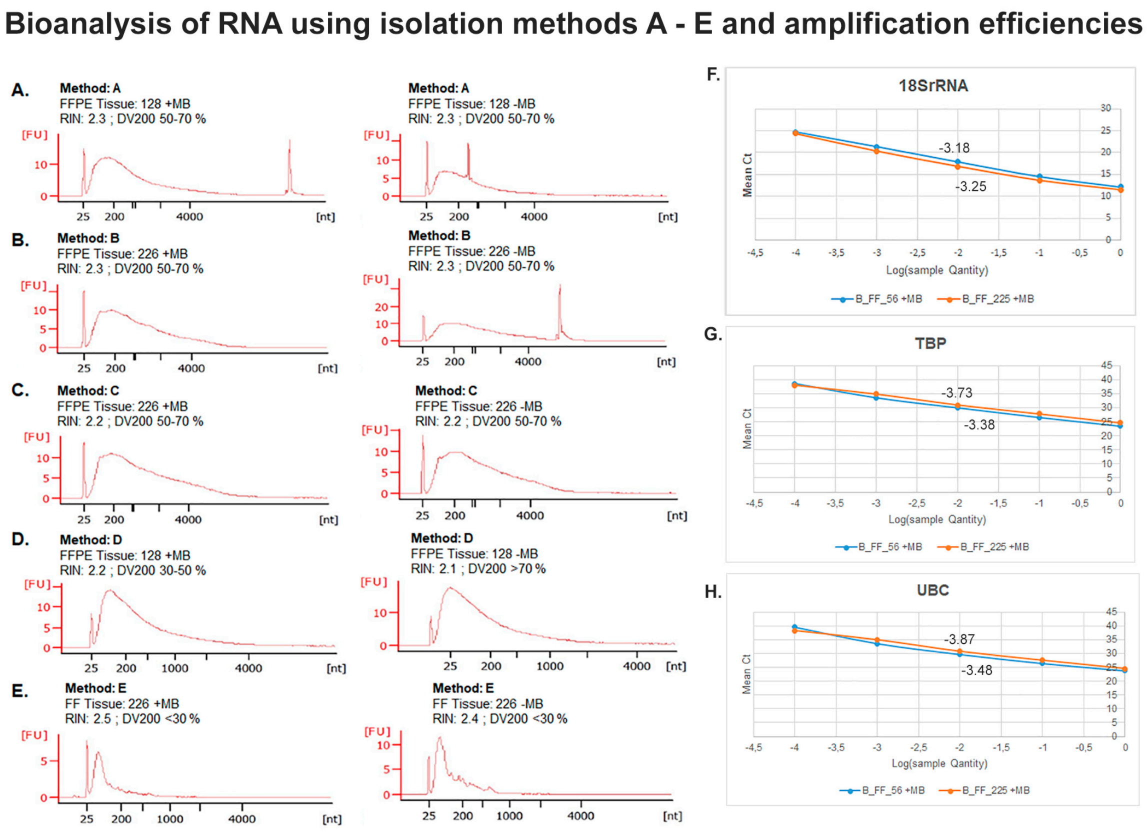

Figure S2. Bioanalysis of RNA from isolation methods and amplification efficiencies. A-E show chromatograms from RNA isolated from FFPE and FF tissues with Method A-E with or without MB, including the RIN and DV200 values for chosen examples; F-G: Two different MIBC RNAs were isolated using Method B with MB and were serially diluted to 1: 10.000. QPCR of *18SrRNA*, *TBP* and *UBC* was performed and standard curves created, which were used to calculate slopes (numbers above/below the curves).

Table S1. Top forward and bottom reverse primer for genes.

Gene name	TF (5′-3′)	BR (5′-3′)
5SrRNA	CCTGAACGCGCCCGATCT	TACAGCACCCGGTATTCCCA
5.8SrRNA	AGCGGTGGATCACTCGGC	CAAGTGCGTTCGAAGTGTCGA
18SrRNA	GCAATTATTCCCCATGAACG	GGCCTCACTAAACCATCCAA
GUSB	ACGTGGTTGGAGAGCTCATTTG	GTTGTCTCTGCCGAGTGAAGA
TBP	GAAGTTGGGTTTTCCAGCTAAGT	ACTAAATTGTTGGTGGGTGAGC
UBC	GAAGATGGTCGTACCCTGTCT	GTCTTGCCAGTGAGTGTCTTC

Left to right columns: Gene name; top forward (TF) and bottom reverse (BR) primer nucleotide sequences for qPCR.

Table S2. Thirty-four reference genes and six MIBC subtyping target genes.

Gene name	Accession number	Gene Type
ACTB	NM_001101.5	Reference gene
ALAS1	NM_000688.6	Reference gene
ATP5F1B	NM_001686.4	Reference gene
B2M	NM_004048.4	Reference gene
CFL1	NM_005507.3	Reference gene
CYC1	NM_001916.5	Reference gene
EIF4A2	NM_001967.4	Reference gene
GAPDH	NM_002046.7	Reference gene
GUSB	NM_000181.4	Reference gene
HMBS	NM_000190.4	Reference gene
HNRNPL	NM_001533.3	Reference gene
HPRT1	NM_000194.3	Reference gene
HSP90AB1	NM_001371238.1	Reference gene
PCBP1	NM_006196.4	Reference gene

PGK1	NM_000291.4	Reference gene
PPIA	NM_021130.5	Reference gene
RBCK1	NM_006462.6	Reference gene
RER1	NM_007033.5	Reference gene
RPL13	NM_000977.4	Reference gene
RPL29	NM_000992.3	Reference gene
RPL37A	NM_000998.5	Reference gene
RPLP0	NM_001002.4	Reference gene
RPS23	NM_001025.5	Reference gene
SDHA	NM_004168.4	Reference gene
TBP	NM_003194.5	Reference gene
TFRC	NM_003234.4	Reference gene
TSFM	NM_001172696.2	Reference gene
TMEM199	NM_152464.3	Reference gene
TUBB	NM_001293212.2	Reference gene
UBB	NM_018955.4	Reference gene
UBC	NM_021009.7	Reference gene
UBE2D2	NM_003339.3	Reference gene
WDR55	NM_017706.5	Reference gene
YWHAZ	NM_145690.3	Reference gene
KRT5	NM_000424.4	Basal target gene
KRT14	NM_000526.5	Basal target gene
CD44	NM_000610.4	Basal target gene
KRT20	NM_019010.3	Luminal target gene
GATA3	NM_001002295.2	Luminal target gene
FOXA1	NM_004496.5	Luminal target gene

Table S3: Reference genes for BLCA / MIBC.

cancer	cohort [n] & tissues	controls [n] & tissues	RNA isolation	Analysis method	reference genes tested	best reference genes	literature reference
BLCA (transitional)	14 (FF)	matched	RNeasy Mini	qPCR	9	SDHA, **TBP**	53
BLCA (transitional)	35 (FF)	35 (matched)	Trizol	qPCR	18	RPS23, TPT1, RPS13	54
BLCA/ MIBC	28 (FFPE)	none	Roche FFPE	qPCR	20 (compared to TCGA)	**UBC**, RPL0, HMBS, **GUSB, TBP**	55
BLCA/ MIBC	29 (FFPE)	5 (bladder)	Promega-FFPE + MB	RNA hybrid.	34	**UBC, GUSB,** EIF4A2, WDR55, **TBP**	this study

Thirty-four reference genes and six MIBC subtyping target genes with NCBI Accession numbers (please see **Table S2**).
